# Medical News and Miscellany

**Published:** 1890-05

**Authors:** 


### Medical News and Miscellany

DR. A. M. WOMACK has removed from Mt. Vernon to Peoria, Hill County.

Dr. W. A. McGEHEE has removed from Uvalde to Alpine, Brewster county.

Dr. W. M. TERRELL has removed from Fort Graham to Farmer, Young county.

Drs. E. J. WARD and B. M. Worsham have formed a co-partnership at Waxahachie.

---

DR. J. W. SMITH, of Pilot Point, after taking in the American Medical Association meeting, at Nashville, on the 20th, will go to New York to attend the Polyclinic.

---

ATTENTION IS CALLED to the announcement of the St. Louis Medical College, the University of Nashville Medical College, and the University of Pennsylvania Medical College,—all new advertisements in this issue.

---

FOR SALE.—A complete and pleasant home, with good location for physician, mostly country practice, but at county seat of new county. For particulars, apply to F. D. Johns, M. D., Leakey, Texas, or the JOURNAL.

---

CHICAGO POLYCLINIC.—The one hundred and twenty-seven physicians who attended the recent course at this institution were so impressed with the thoroughness and marked ability of the course of lectures on abdominal and pelvic surgery by Drs. Senn, Parkes, Bellfield and Fenger, that they adopted and published a set of strong commendatory resolutions, setting forth the opinion thet it was the best and most thorough course ever delivered in America, and asking that it be repeated. Good!

---

THE LONE STAR MEDICAL ASSOCIATION (of colored physicians) will hold their fifth annual meeting in Galveston, June 3, proximo. Dr. J. H. Wilkins, of Galveston, is President, and Dr. M. J. Snowden, of Marshall, is Secretary. Their program is out, and eighteen papers are down for the session, embracing a wide range of subjects. We have been present at one of their meetings, and confess to no little surprise at the amount of intelligence and learning exhibited by some of the members in discussing medical topics. Our colored brethren are striving to improve themselves, and this, with their very modest behavior in not attempting to force themselves upon the white physicians, entitles them, in our judgment, to aid and encouragement at the hands of the people and of the profession. Their decent behavior is in strong contrast to the presumption displayed by certain so-called physicians, who have demanded (?) equal representation on examining and health boards.

---

THE eleventh quarterly meeting of the Austin District Medical Society will be held in its elegant hall in Austin, on Thursday, 19th of June, prox.

Among the papers to be read and discussed will be one by Dr. W. A. Morris, on "Every Day Medical Ethics," by special appointment at last meeting. There will also be papers on "Bright's Disease," "Fracture of the Head of the Humorus tion," "Dysentery," "Resection of Plantar Cutaneous Nerve for Neuralgia Plantoris," "Perineal Preservation During Parturition," "A Case of Gun-shot Wound through the Abdomen."

There will be several voluntary papers and other matters of importance and interest to engage the Society. Every regular physician accessible to Austin is invited to be present and take part in the proceedings.

The June meeting will be specially attractive on account of the article by Dr. W. A. Morris on the subject of ethics, as well as the other interesting and practical subjects on the programme. For further information write Dr. T. J. Bennett, Secretary, Austin, Texas, for June programme.

---

TEXAS MEDICAL COLLEGE AND HOSPITAL, GALVESTON.—We are in receipt of the eleventh annual announcement and catalogue of the above college. The Session of 1890-91, will begin Wednesday, October 1, 1890, and hold six months; there will be no preliminary course.

Since last session several very important plans have been consummated, which add greatly to the usefulness and prosperity of the school. The great Sealey Hospital has been completed and is in operation. The faculty of the school constitute the medical and surgical staff; the superintendent or surgeon in charge, Dr. Wysong, being a member of the teaching corps. This with the St. Mary's College gives abundant clinical advantages; places it, in fact, on a par with the New Orleans schools, which enjoy exceptional advantages in that respect. Since last session, also, there has been opened a Bacteriological laboratory. The outfit for this important addition was purchased in Europe, and is now in hand and under charge of a teacher whose ability in this line is generally conceded.

The standard of the Texas Medical College and Hospital is high. The course of study is graded, and extends over three years; and it is the purpose of the faculty to adhere to, and fully carry out the curriculum published in this catalogue. The method of instruction consists of so blending didactic lectures, recitations, clinical teaching and laboratory work as to enable the student to make the best possible use of his time.

The JOURNAL would advise students to examine well the advantages offered by this, our home school, before deciding where to matriculate, and to consider the saving in railroad and other expense, going and coming; and should their presence be required at home at any time, they are not exposed to the cost and annoyance of a long journey. Address the Dean, Dr. H. P. Cooke, for a catalogue.

